# Using the Integration of Discrete Event and Agent-Based Simulation to Enhance Outpatient Service Quality in an Orthopedic Department

**DOI:** 10.1155/2016/4189206

**Published:** 2016-03-09

**Authors:** Cholada Kittipittayakorn, Kuo-Ching Ying

**Affiliations:** ^1^Graduate Institute of Industrial and Business Management, National Taipei University of Technology, No. 1, Section 3, Zhongxiao E. Road, Taipei 10608, Taiwan; ^2^Department of Industrial Engineering and Management, National Taipei University of Technology, No. 1, Section 3, Zhongxiao E. Road, Taipei 10608, Taiwan

## Abstract

Many hospitals are currently paying more attention to patient satisfaction since it is an important service quality index. Many Asian countries' healthcare systems have a mixed-type registration, accepting both walk-in patients and scheduled patients. This complex registration system causes a long patient waiting time in outpatient clinics. Different approaches have been proposed to reduce the waiting time. This study uses the integration of discrete event simulation (DES) and agent-based simulation (ABS) to improve patient waiting time and is the first attempt to apply this approach to solve this key problem faced by orthopedic departments. From the data collected, patient behaviors are modeled and incorporated into a massive agent-based simulation. The proposed approach is an aid for analyzing and modifying orthopedic department processes, allows us to consider far more details, and provides more reliable results. After applying the proposed approach, the total waiting time of the orthopedic department fell from 1246.39 minutes to 847.21 minutes. Thus, using the correct simulation model significantly reduces patient waiting time in an orthopedic department.

## 1. Introduction

The concept of improving the quality of healthcare service has been repeatedly discussed in recent years. Healthcare providers try to provide patients with better service and excellent treatment. Patients who are satisfied with their service experience are more likely to come back to the hospital in the future [1]. Many studies have regarded patient waiting time as a significant component of patient satisfaction/dissatisfaction [2–7]. Bernhart et al. [8] presented the relative importance of various patient satisfaction factors, finding the service waiting time to be one of the key factors. Patient satisfaction is an important indicator of healthcare outcomes and plays a key role in improving healthcare service quality to attract patients [9].

Patient satisfaction and patient waiting time are important factors in the field of healthcare service. A long waiting time reflects negatively on the quality of the hospital and cripples its competitive advantages [10]. Outpatient waiting time means the time spent by an outpatient in a queue waiting to be served. Throughput time is the amount of time required for a patient to pass through a hospital process. The throughput time includes process time, service time, move time, and waiting time. Utilization rate means the percentage of time a doctor spends doing diagnosis. A direct way to improve patient satisfaction and service quality is to reduce the waiting time. Groome and Mayeaux Jr. [7] explained some factors that affect waiting time, including arrival time, failure to show up for appointments, consultation time, and registration time. Bailey [11] and Welch [12] proposed a single-block/individual system in which the best scheduling policy for patient waiting time is to place two patients at the beginning of the period and then schedule patients evenly over the intervals based on average service time. Wijewickrama and Takakuwa [13] analyzed the long-waiting-time problem in outpatient clinics with a mixed-type registration, using the simulation approach to test four scheduling rules in comparison with the original case. They found that implementing the rule that “first priority is given to shorter processing of patients for consultation with a physician” can achieve the best waiting-time performance. Su and Shih [14] applied the simulation approach to test four assumed models, such as changing patient sequencing and assigning an interval time for scheduled patients. Reynolds et al. [15] tested eight cases to determine how different numbers of doctors and nurses affect waiting time. Baril et al. [16] applied a simulation model to improve performance at an outpatient orthopedic clinic, focusing on the relationships and interactions among patient flows, resource capacity (number of consulting rooms and number of nurses), and appointment scheduling rules. In short, there are different ways to improve patient waiting time amongst which healthcare providers should select the approach that best fits their situation.

Operation-management tools, which are well known in industrial engineering, are being effectively used in the healthcare industry for enhancing both the use of limited resources and system efficiency [17–19]. Recently, the use of computing simulation for achieving a more effective decision-making has exhibited a rising trending [20]. Computer simulation uses computer software to simulate an abstract model of a specific system representing real-world situations. Computer simulation was applied to hospital systems in 1979 to improve the scheduling of staff members [21]. Rohleder et al. [2] used a simulation model to identify certain elements, such as staffing level, patient scheduling, and promptness of service that could reduce patient waiting time at an orthopedic outpatient clinic. Reilly et al. [22] proposed a delay-scheduling model for patients in a walk-in clinic and applied computer simulation to evaluate the clinical performance with different physician staffing patterns and different rules for delay scheduling.

DES is a computer-based methodology that provides an intuitive and flexible approach for representing complex systems. It allows users to estimate the impact of operational changes before expending resources to implement those changes [23]. A DES model can represent the patient visit process, identify process bottlenecks, and adjust resource allocation, without disturbing the actual system [24]. Gul and Guneri applied a DES model to determine the optimal staff level to reduce the patient average length of stay (LOS) in an emergency department, as well as to improve patient throughput and utilization of resources [25]. Lu et al. [26] employed DES to improve outpatient service quality in an orthopedic surgery department. Kim et al. [27] improved a mental health clinic design by using DES.

Entities built into a DES model are typically simple, reactive, and limited in decision-making [28]. Moreover, human capabilities such as multitasking need to be used to set up a validated model [29]. Thus, using only DES is insufficient to model human behavior since the possible path of the entity is predetermined in a DES model [30]. ABS has been proposed to model the human discretion factor in a simulation model [31]. It is a new approach for modeling systems of autonomous, interacting agents [32]. An agent can be described as an autonomous entity that makes decisions based on a set of rules [33]. In the system, agents communicate with one another; they adapt and change their behavior based on the outcome of the interaction [34]. Crooks and Hailegiorgis [35] developed ABS further to explore the spread of cholera. They modeled the spread of cholera by explicitly representing the interaction between humans and their environment.

In outpatient clinic modeling, behaviors related to patients and healthcare providers can be modeled to investigate patient flow, in order to improve waiting time. Aburukba et al. [36] used a distributed multiagent approach to model an intelligent dynamic scheduling solution in advertisement. They believed that the agent-based model is appropriate due to its ability to support both dynamic behavior and distributed structure. Hutzschenreuter et al. [37] demonstrated an agent-based simulation and evaluation tool for patient admission scheduling, with the aim of achieving the efficient use of hospital resources through the combination of different profiles of resource use.

The main aim of this work is to improve clinical services by reducing patient waiting time in an orthopedic department. The integration of DES and ABS is used to determine the optimal scheduling for each consultation session. To the best of our knowledge, this paper is the first study applying the integration of DES and ABS models to solve the operational problem in a hospital. The integration of DES and ABS can take advantage of both approaches [38]. DES provides an environment for agents as well as work rules. The orthopedic outpatient clinic environment can be constructed by using DES for orthopedic outpatient flow and by applying ABS for human decision-making.

The rest of the paper is organized as follows.  Section 2 describes the patient flow at the outpatient clinic of an orthopedic department and explains a simulation model.  Section 3 shows the results after applying the proposed approach.  Section 4 discusses the results before and after applying the proposed approach. Finally, Section 5 presents our conclusions.

## 2. Methods

The community hospital in this research is a 689-patient-bed medical center with more than 20 clinical departments and has approximately seven hundred employees, including one hundred physicians, three hundred nurses, and three hundred staff members. The patient volume in the orthopedic department at this community hospital is over 5500 per year and faces the challenge of increasing patient visits. The department, which has nine doctors, is divided into three different teams: hand and foot, trauma, and sports. Current data show that the outpatients on average spend only about 14 minutes to get serviced but almost two hours (104 minutes) waiting in line; waiting time accounts for over 88% of the total process time.

### 2.1. Description of the Outpatient Orthopedic Department

**Figure 1:**
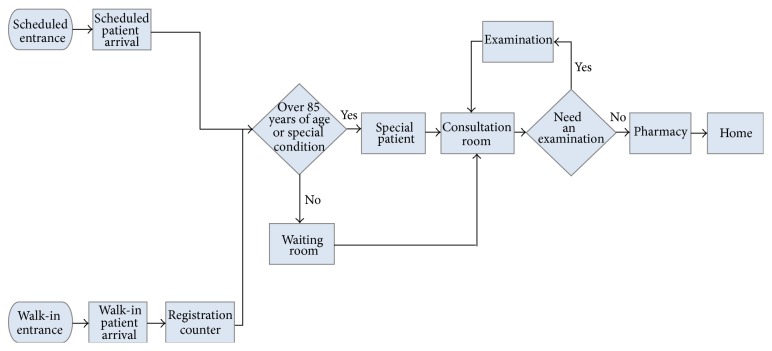
Patient flow at the outpatient clinic of the orthopedic department.

The orthopedic department operates from 8:30 a.m. to 10:00 p.m. on weekdays and has 12 consultation sessions per week. The five consultation sessions are treated by doctors from the sports team, the four consultation sessions are treated by doctors from the trauma team, and the three consultation sessions are treated by doctors from the hand and foot team. The types of patients for each consultation session depend on the doctor team. For instance, if the session is seen by a doctor from the trauma team, then most patients in this session would be injured patients. Based on the observations of this department, we formulate a patient flowchart, as shown in Figure 1. The following processes are typical for an orthopedic outpatient visit:

- A walk-in patient stands in line for registration; during registration, basic information such as name, birth date, and identity number is collected.
- After registration, the walk-in patient goes to the orthopedic clinic and waits for consultation.
- A scheduled patient can go directly to the clinic and wait for consultation.
- Both types of patients can be seen according to their registered numbers.
- For current scheduling policy, odd numbers are assigned to walk-in patients and even numbers are assigned to scheduled patients (a doctor will see one walk-in patient and then one scheduled patient).
- The orthopedic department has a special policy: patients who are over 85 years of age or have a special condition have first priority to see a doctor. In this study, we define these patients as “special patients.”
- Appointments for patients who arrive late will be postponed until the following three patients have been seen.
- After a patient consults a doctor, it is decided whether the patient needs to have a medical examination. If so, the patient goes for the examination and will return to see the doctor again. The doctor will prescribe medicine if required. The patient then receives the medicine and leaves the hospital or receives a prescription, picks up the drug, and leaves the hospital.

### 2.2. System Analysis

The department's operations and details of each consultation section were investigated through interviews with staff members, close observations of the department's daily operations, and collecting the hospital database. Focus groups were formed, including orthopedic surgeons, nurses, healthcare assistants, and radiologists.

**Figure 2:**
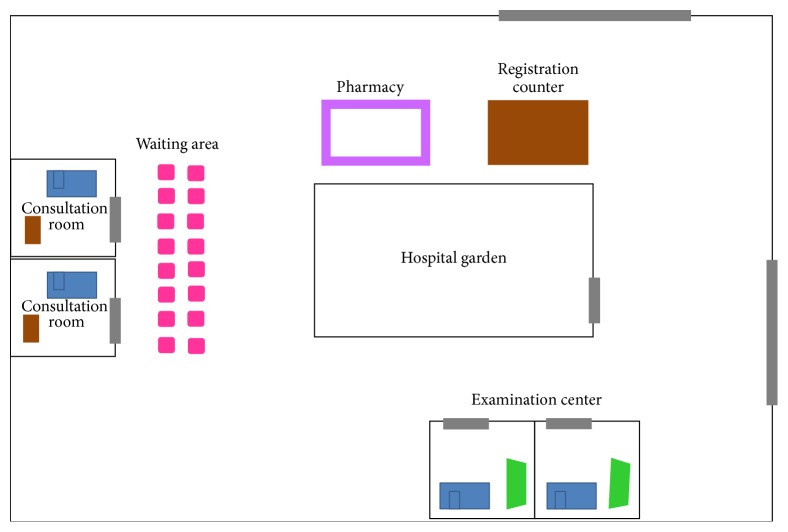
Orthopedic department layout.

The orthopedic department contains different zones, including registration area, waiting area, treatment area, examination room, and pharmacy. The department shares the examination room and pharmacy with other departments.  Figure 2 shows the layout of the department.

All data were collected from Monday to Friday from the orthopedic department over a two-month period (June to July) by hospital staff. Different orthopedic department areas were observed with the purpose of analyzing and taking notes about how the different processes take place, and data were collected on registration time, patient queues, clinic start time, consultation period, clinic end time, arrival time, consultation time, late rate, no-show rate, examination time, and examination rate. The collected data were formed into two groups. The June data were denoted as model-building data and were used to build a simulation model. The July data were used to validate the simulation model. All data were analyzed statistically and used to construct a simulation model.

### 2.3. Orthopedic Department Modeling

Developing a simulation model consists of the following steps: describing a problem, formulating a problem, collecting and processing real system data, formulating and developing a model, verifying and validating a model, documenting a model for future use, selecting appropriate experimental design, establishing experimental conditions for runs, performing simulation runs, and interpreting results [39]. The orthopedic department model defined by this study is an integration of ABS and DES, incorporating a population of simulated real-world outpatient-use data with a discrete event simulation of an orthopedic department. We observe that the orthopedic department processes consist of continuous changes, so that when we analyze these processes, it is better to divide a continuous process into discrete parts to simplify the analysis. In this case, DES is the best choice when the system under analysis can naturally be described as a sequence of operations.

Since the orthopedic department is a dynamic environment with full human interactions, human behaviors affect the outcome of those interactions. The entities of an orthopedic department, such as doctors, nurses, and administration staff, are humans with emotions and reasoning abilities. These agents adapt and change their behavior during the simulation. ABS allows us to explore the system, which has a natural representation consisting of interacting agents. In this situation, it is best to apply agent-based modeling to capture emergent phenomena. For modeling purposes, patients are considered as static objects. This study develops a simulation model using AnyLogic 7 to represent the patient flow in the orthopedic department. We choose AnyLogic 7, because it supports DES and ABS and allows us to efficiently combine it with other modeling approaches. The simulation of the current system without any changes is run as the case-based model. The results of the simulation provide a baseline for comparing operational changes.

### 2.4. Simulation and Implementation

The simulation model is implemented in AnyLogic 7. This is the only simulation tool that supports all of the most common simulation methodologies, such as system dynamics and discrete event and agent-based modeling, and enables the user to capture the complexity and heterogeneity of business, economic, and social systems to any desired level of detail.

The current version of the simulator includes the registration counter, waiting room, consultation room, examination center, and pharmacy. Through the information obtained during interviews carried out with the orthopedic staff, this study identifies two kinds of agents: active agents and passive agents [40]. The active agents in this simulation are orthopedic outpatients, doctors, nurses, healthcare assistants, radiologists, biomedical scientists, and administration staffs. The passive agents represent solely reactive aspects of the system, such as the patient information system, examination center, and loudspeaker system.

In the simulation model, orthopedic outpatients arrive at the orthopedic clinic by their own means and go to the registration counter. In the simulation the input information is read from a text file within the data given by the hospital. Once the registration process has been carried out, the patients go to the waiting area. Patients who are over 85 years of age or have a condition can be seen by a doctor first. The patients' scheduling changes depending on the numbers of walk-in patients and scheduled patients inside the waiting area. Once the patient is seen by a doctor, the doctor decides whether the patient needs an examination. After seeing a doctor, the patient goes to the pharmacy and then leaves the hospital.

## 3. Results

### 3.1. Model Validation

The model validation ensures that the model correctly represents the real world. The simulation model is validated by comparing data generated by the model and data collected from the orthopedic department.  Table 1 shows confidence intervals of the simulation outputs at the 95% (*α* = 0.05) confidence level and the actual values obtained from the collected data. The comparison verifies that, for waiting time, throughout time, and utilization, there are no significant differences between the results obtained using the simulation and those that occurred in the real system. We conclude that the model is truly representative of the existing environment. Therefore, the validated model can be used for subsequent analysis.

### 3.2. Main Results

This research applies the integration of DES and ABS to an orthopedic clinic. The different consultation sessions yield different results, which are shown in Table 2. To further verify the effectiveness of the proposed approach, the study performs the paired *t*-test on the average real-world waiting time to compare the waiting times before and after applying the approach. At the confidence level *α* = 0.05, the results in Table 3 clearly confirm the superiority of the proposed approach.

## 4. Discussion

The main objective of this study is to reduce the outpatient waiting time in the orthopedic department. After applying the proposed approach, the results show that the waiting time dropped significantly: for consultation session 1, the waiting time fell to 39.28 minutes (32.47%); for consultation session 2, the waiting time fell to 43.72 minutes (35.16%); for consultation session 3, the waiting time fell to 21.97 minutes (27.89%); for consultation session 4, the waiting time fell to 16.75 minutes (26.35%); for consultation session 5, the waiting time fell to 22.27 minutes (27.64%); for consultation session 6, the waiting time fell to 35.67 minutes (30.51%); for consultation session 7, the waiting time fell to 23.85 minutes (28.60%); for consultation session 8, the waiting time fell to 55.97 minutes (38.23%); for consultation session 9, the waiting time fell to 22.8 minutes (29.34%); for consultation session 10, the waiting time fell to 37.79 minutes (31.78%); for consultation session 11, the waiting time fell to 56.62 minutes (37.65%); for consultation session 12, the waiting time fell to 22.5 minutes (26.64%). The average waiting time after applying the proposed approach of 12 consultation sessions fell to 33.27 minutes (32.03%).

It is obvious from Tables 2 and 3 that the proposed approach is very effective for improving clinical services by reducing patient waiting time in the orthopedic department. These results underscore the benefits of modeling operational changes before implementation, particularly under a resource limitation situation. This study shows that simulation models can be useful decision-support tools for healthcare provider management, not only for waiting time reduction in an orthopedic department, but also for the hospital as a whole.

## 5. Conclusions

Service quality, which always influences hospital patient satisfaction, has recently become an important index in the healthcare field. Our previous study has shown that waiting time is a key performance index of patient satisfaction. Computer simulation is an efficient approach to study such a complex system. The increasing interest in the integration of simulation approaches may be explained by the increasingly complex nature of the problems being faced. Several problems involve interacting elements of a different nature. Thus, modelers face the choice of identifying the best single paradigm or adopting multiple paradigms, such as an integration of the simulation approach and applying it to the whole system.

In this paper we have integrated DES and ABS simulation models to evaluate proposed strategies applied toward an orthopedic department so as to reduce patient waiting time. The integration of DES and ABS allows us to utilize the advantages of both simulations. DES helps us to understand the orthopedic department processes and to replicate the orthopedic department system, whereas ABS allows us to consider the variation in individual behavior in order to model a situation with interdependencies between work entities. The simulation model herein correctly emulates the patient flow in the orthopedic department and can be used to analyze the effects of potential improvement policies. The research results indicate that the proposed approach achieves a considerable reduction in waiting time. Moreover, the reduction in waiting time does not need any additional resources.

Although our results suggest that the integration of DES and ABS can improve the waiting time in an orthopedic clinic, there are several important limitations to discuss. First, since this research provides only one example, more case studies implementing the model are needed for external validity. Second, the proposed simulation model only generates a method of evaluating a solution but does not generate solutions themselves. Finally, the proposed simulation model does not yield an answer. It merely provides a set of the system's responses to different operating conditions, and so the results need to be well interpreted and understood before any changes are implemented.

The approach of this research not only is applicable to the orthopedic department at this hospital but also can be applied to reduce patient waiting time at other orthopedic departments nationwide. Furthermore, the results can be helpful for other hospital departments in addition to orthopedics.

## Figures and Tables

**Table 1:** Validation of simulation model by a comparison between simulated and collected data.

	Simulation model	Confidence interval	Collected data	Difference (%)
Consultation session variable 1				
Average waiting time (minute)	124.36	[105.08, 143.64]	121.77	2.083
Average throughput time (minute)	132.54	[120.27, 144.81]	134.12	1.192
Average utilization (%)	92.16	[76.26, 106.06]	93.51	1.46
Consultation session variable 2				
Average waiting time (minute)	122.57	[100.66, 144.48]	125.98	2.78
Average throughput time (minute)	129.92	[118.53, 141.31]	131.45	1.18
Average utilization (%)	88.56	[74.54, 102.58]	84.97	1.79
Consultation session variable 3				
Average waiting time (minute)	77.75	[48.84, 106.68]	75.41	3.00
Average throughput time (minute)	85.47	[71.45, 99.49]	86.68	1.42
Average utilization (%)	85.63	[54.95, 116.31]	82.78	3.33
Consultation session variable 4				
Average waiting time (minute)	65.15	[49.37, 80.93]	66.13	1.50
Average throughput time (minute)	72.31	[50.4, 94.22]	74.14	2.53
Average utilization (%)	91.53	[76.19, 106.87]	90.23	1.42
Consultation session variable 5				
Average waiting time (minute)	81.36	[61.64, 101.08]	79.65	2.10
Average throughput time (minute)	89.37	[71.23, 107.51]	90.98	1.80
Average utilization (%)	87.67	[72.24, 103.1]	86.36	1.49
Consultation session variable 6				
Average waiting time (minute)	116.3	[107.01, 125.59]	117.24	0.81
Average throughput time (minute)	137.0	[126.92, 147.08]	138.38	1.01
Average utilization (%)	91.7	[70.66, 112.74]	89.68	2.20
Consultation session variable 7				
Average waiting time (minute)	85.1	[66.79, 103.41]	86.76	1.95
Average throughput time (minute)	102.0	[86.97, 117.03]	103.51	1.48
Average utilization (%)	92.6	[88.66, 96.54]	92.57	0.03
Consultation session variable 8				
Average waiting time (minute)	145.3	[137.41, 153.19]	144.12	0.81
Average throughput time (minute)	156.0	[137.33, 174.67]	158.98	1.91
Average utilization (%)	95.5	[78.85, 112.15]	93.97	1.60
Consultation session variable 9				
Average waiting time (minute)	75.3	[59.43, 91.17]	74.12	1.57
Average throughput time (minute)	90.0	[72.66, 107.34]	88.32	1.87
Average utilization (%)	91.8	[66.47, 117.13]	94.21	2.63
Consultation session variable 10				
Average waiting time (minute)	122.1	[115.08, 129.12]	122.97	0.71
Average throughput time (minute)	130.63	[117.6, 143.66]	132.45	1.39
Average utilization (%)	89.84	[75.2, 104.48]	88.41	1.59
Consultation session variable 11				
Average waiting time (minute)	151.51	[136.08, 166.94]	154.01	1.65
Average throughput time (minute)	159.83	[145.63, 174.03]	157.63	1.38
Average utilization (%)	96.09	[74.09, 118.09]	98.34	−2.34
Consultation session variable 12				
Average waiting time (minute)	82.82	[72.5, 93.14]	83.68	1.04
Average throughput time (minute)	95.52	[74.39, 116.65]	97.66	2.24
Average utilization (%)	94.21	[71.71, 116.71]	91.96	2.39

**Table 2:** Waiting time improvement for the orthopedic department.

Consultation session	Average real-world waiting time	Average waiting time after applying the proposed approach	Waiting time improvement (%)
1	2:00:59^*∗*^	1:21:42	32.47
2	2:04:21	1:20:38	35.16
3	1:18:46	0:56:48	27.89
4	1:03:34	0:46:49	26.35
5	1:20:34	0:58:18	27.64
6	1:56:54	1:21:14	30.51
7	1:23:24	0:59:33	28.60
8	2:26:24	1:30:26	38.23
9	1:17:42	0:54:54	29.34
10	1:58:55	1:21:08	31.83
11	2:30:23	1:33:46	37.65
12	1:24:27	1:01:57	26.64

^*∗*^hh:mm:ss.

**Table 3:** Paired *t*-tests performed on average real-world waiting time (after applying the approach versus before applying the approach).

After applying the approach	Before applying the approach
Difference	1995.5
Dof	11
*t*-value	−8.347
One-tail significance	<0.0001
